# Deubiquitinase USP7 Regulates Neutrophil Extracellular Trap Formation and Inflammation in Lipopolysaccharide‐Treated Mice Through ICAM‐1 Expression

**DOI:** 10.1002/kjm2.70133

**Published:** 2025-10-31

**Authors:** Hui Xu, Qi Wang, Jing‐Xian Fan, Yong‐Fu Xu, Jiao Yu

**Affiliations:** ^1^ Department of Emergency Shanghai Ninth People's Hospital Affiliated to Shanghai Jiaotong University School of Medicine Shanghai China

**Keywords:** intercellular adhesion molecule‐1, neutrophil extracellular traps, sepsis, ubiquitin‐specific peptidase 7

## Abstract

Sepsis is typified by organ failure due to an unchecked host reaction to infection. This study aims to explore the mechanism of ubiquitin‐specific peptidase 7 (USP7) in sepsis with the involvement of intercellular adhesion molecule‐1 (ICAM‐1). A sepsis model was established using lipopolysaccharide (LPS) induction in WT and USP7^−/−^ mice, and various assays were conducted to evaluate survival rates, organ damage, inflammatory markers, and protein interactions. The results revealed that USP7 expression increased in LPS‐treated WT mice, and its interaction with ICAM‐1 stabilized ICAM‐1 through deubiquitination. USP7 knockout significantly elevated survival rates of septic mice. USP7 knockout reduced pulmonary inflammation, neutrophil extracellular trap (NET) formation, and myeloperoxidase and Cit‐H3 levels in septic mice. Moreover, USP7 knockout lowered the levels of organ injury markers (creatine kinase‐MB [CK‐MB], troponin‐I, and blood urea nitrogen [BUN]), liver enzymes (ALT and AST), and inflammatory markers (TNF‐α, IL‐1β, IL‐6, and IL‐8). Co‐culture of bone marrow‐derived macrophages (BMDMs) from WT mice with ICAM‐1+ neutrophils elevated levels of TNF‐α, IL‐1β, and IL‐6. These findings suggest that USP7 plays a critical role in driving sepsis‐induced NET formation and inflammation by stabilizing ICAM‐1. Targeting USP7 may represent a potential therapeutic approach to mitigate sepsis‐related inflammation and organ damage.

## 1 Introduction

Sepsis is a potentially fatal illness typified by organ failure ascribed to an unchecked host reaction to infection, and its pathological state is commonly characterized by a significant drop in blood pressure, which leads to decreased tissue perfusion and oxygen delivery, ultimately causing hypoxia, a hallmark feature of shock [1]. Sepsis is primarily triggered due to infections of the respiratory, gastrointestinal, genitourinary, and skin and soft tissue systems [2]. It falls into two concurrent phases, namely an early phase of immune activation followed by a phase of chronic immunosuppression, provoking immune cell death [3]. As an essential component of the innate immune response, neutrophil extracellular traps (NETs) are formed upon the release of decondensed chromatin and other nuclear materials through a process known as NETosis. While NETs play a crucial role in trapping and neutralizing pathogens, their excessive formation can be detrimental under conditions like sepsis since their overproduction leads to tissue damage [4]. Excessive NETs can further cause inflammation and organ injury, resulting in sepsis progression [5]. Hence, understanding molecular mechanisms of NET formation and inflammatory responses in sepsis is beneficial to the development of new therapeutic strategies for sepsis.

Ubiquitin‐specific proteases (USPs) are a family of deubiquitinating enzymes that are important to the fate of proteins and the transduction of pathways by cleaving ubiquitin chains from target proteins, which are indispensable for various physiological processes and have a profound impact on immune responses and inflammatory conditions [6]. USP7 is involved in regulating multiple key proteins, including tumor suppressors, transcription factors, epigenetic modulators, DNA repair proteins, and immune response regulators [7]. USP7 also plays a pivotal role in stabilizing molecules that promote angiogenesis and metastasis, and pharmacological inhibition of USP7 has garnered attention because of its therapeutic value in cancer and other diseases [8]. Furthermore, USP7 has been implicated in sepsis‐induced myocardial injury and cardiomyocyte pyroptosis [9]. Intercellular adhesion molecule‐1 (ICAM‐1) is a cell surface glycoprotein well known for recruiting leukocytes from circulation to inflammatory sites [10]. It is robustly upregulated on epithelial and immune cells after inflammatory stimulation [11]. Importantly, a prior study elucidated that ICAM‐1 participated in neutrophil activation and NET formation during sepsis and that ICAM‐1‐expressing neutrophils produced excessive NETs, contributing to Rho‐activated inflammation and ultimately exacerbating the pathological state of sepsis [12]. Nevertheless, little has been reported on the relationship between USP7 and ICAM‐1. We constructed USP7 knockout mice to validate the effects of USP7 on the survival rate, NET release, multiple organ dysfunction, and inflammatory response in septic mice. We also validated that USP7 affected the inflammatory response by regulating ICAM‐1 expression through in vitro silencing of USP7. At the same time, we explored the relevant mechanisms of USP7 regulation of ICAM‐1 expression through in vitro experiments. Accordingly, the present study aimed to investigate whether USP7 modulated NET formation and inflammation during sepsis via ICAM‐1.

## 2 Materials and Methods

### 2.1 A Mouse Model of Sepsis

Healthy C57BL/6 wild‐type (WT) mice (aged 6–10 weeks; SLAC Laboratory, Shanghai, China) and USP7 CIRP−/− (USP7^−/−^) mice (Cyagen Biosciences, Suzhou, China) were kept under controlled conditions (a temperature of 25°C–27°C and a humidity of 45%–50%) with ad libitum access to food and water. WT and USP7^−/−^ mice were randomized into two groups (six mice per group). After adaptive feeding, mice were subjected to modeling treatment. The lipopolysaccharide (LPS; SIGMA, St Louis, MO, USA) group of mice was injected intraperitoneally with LPS at a dose of 10 mg/kg [13, 14], while the phosphate‐buffered saline (PBS) group of mice received an intraperitoneal injection of an equivalent dose of PBS.

Mice were randomized into the following four groups: WT control, WT LPS, USP7^−/−^ control, and USP7^−/−^ LPS, with six mice per group. Mice were euthanized using 4% pentobarbital sodium. All animal experiments rigorously followed “Guidelines on Humane Treatment of Laboratory Animals” issued by the Ministry of Science and Technology of China and Guide for the Care and Use of Laboratory Animals issued by NIH. Additionally, the study strictly adhered to the principle of completing the experiment with the minimum number of animals and minimizing the suffering of the experimental animals.

### 2.2 Hematoxylin and Eosin (HE) Staining

Lung tissues were fixed, washed, and dehydrated in ethanol before embedding in paraffin and sectioning. The sections were stained with hematoxylin for 10 min at room temperature, rinsed with water for 30 min, and dipped in 1% hydrochloric acid before another wash. Subsequently, sections were observed under a fluorescence microscope (Primo Star iLED; Boruisi Technology, Beijing, China) to assess pathological changes after eosin staining (5 min), ethanol dehydration, and neutral gel mounting.

### 2.3 Western Blot

Tissues: tissues were harvested through trypsinization and lysed using enhanced RIPA buffer with protease inhibitors (Boster, Wuhan, China). Serum: Blood samples were collected from each group of mice and centrifuged at 3000*g* for 20 min, and serum was separated and lysed. The bicinchoninic acid Protein Assay Kit (Boster) was employed to measure the protein concentration. Proteins were transferred onto PVDF membranes after being separated using 10% SDS‐PAGE. To avoid nonspecific binding, the membranes were sealed (2 h; room temperature) using 5% bovine serum albumin. The following primary antibodies were used: anti‐USP7 (ab108931; 1:10,000; Abcam, Cambridge, UK), anti‐ICAM‐1 (ab282575; 1:1000; Abcam), anti‐citrullinated histone H3 (Cit‐H3; ab5103; 1:1000; Abcam), and anti‐glyceraldehyde‐3‐phosphate dehydrogenase (GAPDH; ab9485; 1:2000; Abcam). After overnight incubation with primary antibodies at 4°C, the membranes were probed with HRP‐conjugated secondary antibodies (ab205719; 1:2000; Abcam; 1 h; room temperature). The membranes were visualized using ECL reagents (EMD Millipore, USA) and developed with a gel imaging system (iBright CL750, Invitrogen, CA, USA). The grayscale value of protein bands was quantified using ImageJ software, with GAPDH used as a loading control.

### 2.4 Detection of Cell‐Free DNA (cfDNA) and NETs

The Quant‐It PicoGreen dsDNA Assay Kit (Thermo Fisher Scientific, Waltham, MA, USA) was used to quantify cfDNA and NETs in serum under the manufacturer's instructions. The intensity of the fluorescence was measured (emission: 530 nm; excitation: 480 nm), reflecting the quantity of DNA in the sample [15].

### 2.5 Immunofluorescence

NETs in peripheral blood were visualized using immunofluorescence confocal microscopy. The samples were incubated with primary antibodies (1:200; Abcam): anti‐histone H3 (#5103) and anti‐myeloperoxidase (134132), followed by incubation with secondary antibodies (Thermo Fisher Scientific) of polyclonal goat anti‐mouse Alexa Fluor 647 and anti‐rabbit Alexa Fluor 488. DNA was stained with DAPI (Sigma‐Aldrich). Images were acquired using a laser confocal fluorescence microscope (LEXT OLS5100; Olympus Corporation, Tokyo, Japan).

### 2.6 Measurement of Myeloperoxidase (MPO) Activity

MPO activity was measured using an MPO assay kit. A microplate reader (Elx800; Biotek, Winooski, VT, USA) was adopted to detect absorbance at 650 nm, and a standard curve was plotted to calculate the concentration.

### 2.7 Blood Biomarkers of Organ Injury

Following the establishment of the mouse model of LPS‐induced sepsis, blood samples were collected from mice and centrifuged (3000*g*, 20 min) to separate the serum. Serum levels of creatine kinase‐MB (CK‐MB), troponin‐I, blood urea nitrogen (BUN), alanine aminotransferase (ALT), and aspartate aminotransferase (AST) were analyzed using assay kits (Bionovo, Beijing, China) and measured with a semiautomatic biochemical analyzer (Coulter LH 750).

### 2.8 Enzyme‐Linked Immunosorbent Assay (ELISA)

The concentrations of TNF‐α (ab208348, Abcam), IL‐1β (ab197742, Abcam), IL‐6 (ab222503, Abcam), and IL‐8 (ab243016, Abcam) in the culture medium and serum were determined using ELISA Kits following the manufacturer's instructions.

### 2.9 Flow Cytometry

Neutrophil sorting: tissues were prepared into single‐cell suspensions, which were fixed with 2% paraformaldehyde, washed, and blocked for 30 min with PBS encompassing 2% bovine serum albumin. The cell surface was stained with rat anti‐mouse Ly‐6G, FITC‐labeled anti‐mouse MPO, and anti‐rabbit CitH3, with IgG as the secondary antibodies. To avoid intracellular chromatin and enzyme staining, cells were not permeabilized. Data were acquired using a BD LSR Fortessa flow cytometer (BD Biosciences, San Jose, CA, USA) and analyzed with FlowJo software (Tree Star, Ashland, OR, USA). In WT mice, quadrilateral/rectangular gating Ly6G+ neutrophils positive for MPO and CitH3 were chosen as NET+ neutrophils. Neutrophils were treated with sh‐USP7, oe‐USP7 lentivirus, and their negative control (GeneCopoeia, Rockville, MD, USA) [16, 17] at an infection multiplicity of 50, and the lentivirus titer was 8 × 10^8^ TU/mL.

ICAM‐1+ neutrophils: The tissues were processed into a single‐cell suspension, fixed with 2% paraformaldehyde, washed, and then blocked with PBS containing 2% bovine serum albumin for 30 min. The cell surface was stained with rat anti‐mouse Ly‐6G, PE anti‐mouse ICAM‐1, and PE donkey anti‐rabbit IgG. To avoid staining chromatin and enzymes within cells, cells were not permeabilized. After collecting sufficient data using a BD LSR Fortessa flow cytometer (BD Biosciences), the data were analyzed using FlowJo software (Tree Star, Ashland, OR). In dot plot analysis, cells were first gated through forward analysis based on their size, intracellular granularity, and side scatter. Next, neutrophils were gated based on Ly6G‐positive staining. Then, cells were gated based on ICAM‐1 antibody staining according to their surface/extracellular characteristics. ICAM‐1+ neutrophils were identified as Ly6G+ neutrophils positive for ICAM‐1. Other steps were identical to those used for the assessment of NET contents.

### 2.10 Isolation of Bone Marrow‐Derived Macrophages (BMDMs)

Bone marrow was extracted from the femurs of C57BL/6 mice. The mice were euthanized, and the muscles were completely removed from the hind legs. The bones were washed three times with PBS, and the tibia was dissected. The bones were then crushed in a mortar, and the supernatant was attained. To remove solid debris, the supernatant was filtered using a 70 μm nylon cell filter. After centrifugation of the filtrate (10 min, 4°C, 450*g*), the pellet was lysed in 10 mL of red blood cell lysis buffer for 30 s. The sample was then added to 20 mL of Dulbecco's modified Eagle's medium (DMEM; 11965092; Gibco, Waltham, MA, USA) encompassing 10% FBS (10100147; Gibco) and centrifuged (450*g*, 4°C, 10 min). The supernatant was discarded, and the pellet was resuspended in 20 mL of complete DMEM pre‐warmed to 37°C and incubated at 37°C for 4 h. After being harvested, the supernatant was centrifuged (450*g*, 4°C, 10 min), and the supernatant was discarded. The collected cells were cultured in 10 mL of complete DMEM supplemented with 15% L929 cell‐conditioned medium. The cells were filtered through a 40 μm nylon cell filter, and the filtrate was obtained. The collected filtrate (10 mL) was added to 140 mL of complete DMEM supplemented with 15% L929 cell‐conditioned medium and incubated for 3 days (37°C, 5% CO_2_). Then, 10 mL of complete DMEM supplemented with 15% L929 cell‐conditioned medium was added for cell incubation at 37°C with 5% CO_2_. On Day 7, BMDMs were harvested. The ICAM‐1‐ neutrophils and ICAM‐1+ neutrophils were co‐cultured with BMDMs.

### 2.11 Co‐Immunoprecipitation (Co‐IP)

Neutrophils were washed with PBS and homogenized on ice after the addition of lysis buffer. The lysates were then centrifuged at 13,400*g* for 10 min at 4°C, and the supernatant was acquired. The supernatant was pre‐incubated with ImmunoPure Protein A/G Agarose (1 h, 4°C) to eliminate non‐specific binding. Thereafter, the supernatant was cleared with 25 μL of Protein A/G Agarose (4°C). Immunoprecipitation was performed overnight at 4°C using USP7 and ICAM‐1 antibodies. The protein complexes were harvested after the samples underwent a 2‐h incubation with 30 μL of Protein A/G Agarose at 4°C. Protein samples were prepared. To analyze the target proteins, the protein complexes were separated using SDS‐PAGE, electrotransferred to the PVDF membrane using the wet transfer method, and examined using Western blot.

### 2.12 Ubiquitination Level Detection

Cells were treated with 10 μM MG132 (M7449, Sigma‐Aldrich) for 8 h, lysed with RIPA buffer containing 1% SDS, and gently sonicated. The resulting cell lysate was diluted to a final concentration of 0.2% SDS using SDS‐free lysis buffer before IP, and cells were cultured with ICAM1 primary antibody at 4°C. Ubiquitination levels were then analyzed by Western blot.

### 2.13 Protein Stability Detection

To evaluate protein stability, cells were treated with cycloheximide (100 μg/mL, Sigma‐Aldrich). After cycloheximide treatment at 0, 4, and 8 h, cells were then lysed in RIPA buffer (P0013B; Beyotime, Shanghai, China), centrifuged (5000*g*, 10 min), and the proteins were extracted. Cells were lysed in RIPA buffer encompassing 0.1% SDS, washed with the washing buffer, and assessed with Western blot. The Quantity One program (Bio‐Rad Laboratories, Hercules, CA, USA) was used to quantify the grayscale value of protein bands [18].

### 2.14 Statistical Analysis

SPSS version 21.0 (IBM SPSS Statistics, Armonk, NY, USA) was used for statistical analysis. The data were presented as mean ± standard deviation. Two‐group comparisons were performed using the unpaired *t* test, while multiple‐group comparisons were conducted using one‐way analysis of variance (ANOVA), with Tukey's test for post hoc analysis. Repeated measures ANOVA was used for comparisons at different time points. A *p* < 0.05 was considered statistically significant.

## 3 Results

### 3.1 USP7 Deficiency Increases the Survival Rate of LPS‐Treated Mice

**FIGURE 1:**
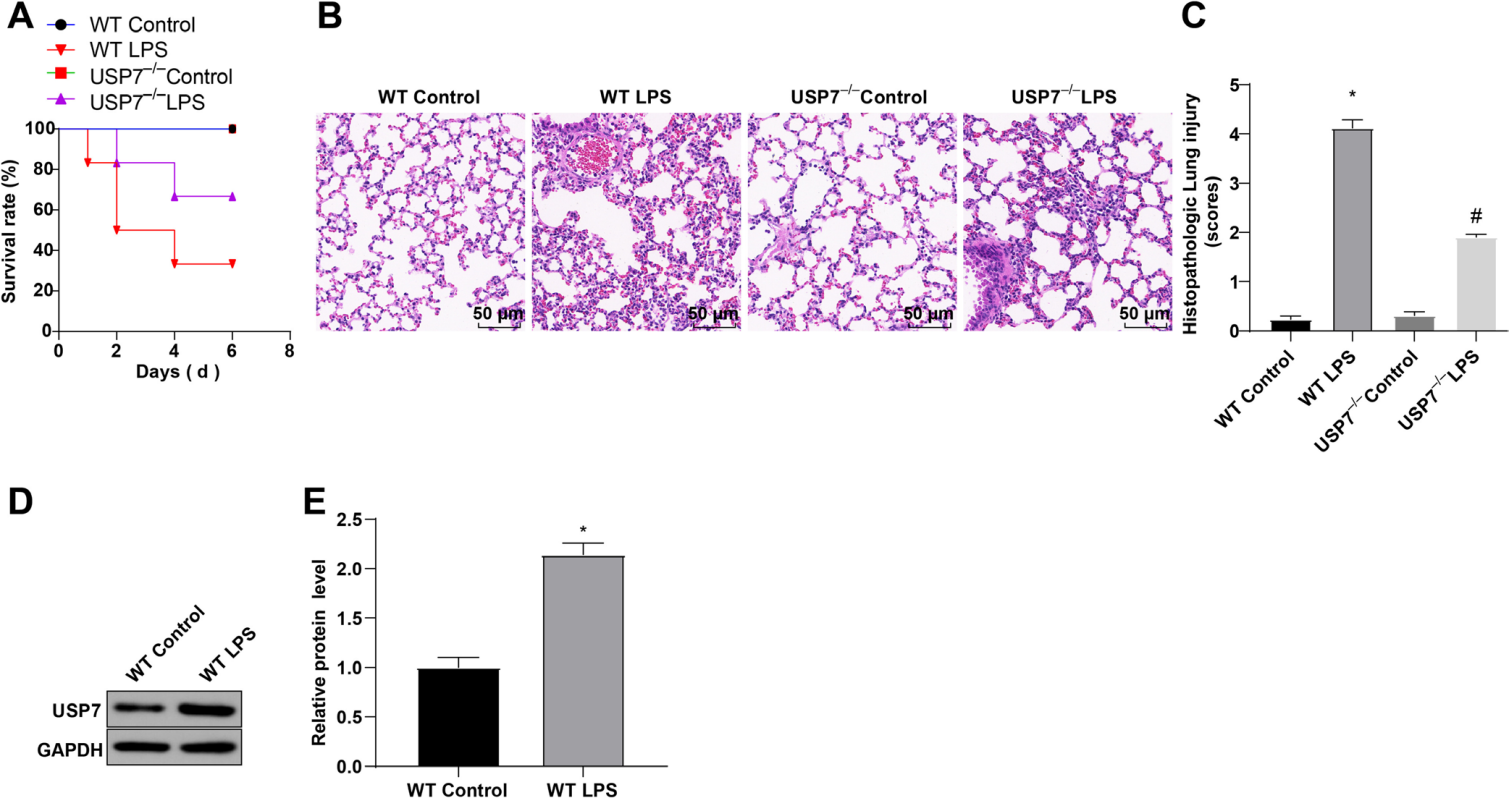
Knockout of USP7 affects survival rate in septic mice. (A) Survival rate of mice in each group. (B) HE staining to detect pathological changes in lung tissue of mice. (C) Histopathological scores according to HE staining. (D, E) Western blot analysis of USP7 expression. *p < 0.05 compared to the WT Control group, ^#^ *p* < 0.05 compared to the WT LPS group; data were presented as mean ± SD; comparisons between groups were performed using one‐way ANOVA; *n* = 6.

To explore the effect of USP7 on sepsis, we developed a mouse model of sepsis following USP7 knockout and measured the survival rates of mice. The findings demonstrated that the survival rate in the USP7^−/−^ LPS group was higher than that in the WT LPS group, whereas the survival rate in the WT LPS group was significantly lower than that in the WT Control group (Figure 1A). Histopathological analysis of lung tissues via HE staining revealed that, compared to the WT Control group, the WT LPS group exhibited increased pathological severity, with vascular congestion, edema, and significant neutrophil infiltration. In contrast, the USP7^−/−^ LPS group showed reduced pathological severity versus the WT LPS group (Figure 1B,C). WB detection results showed that, compared with the WT Control group, USP7 expression in the WT LPS group was significantly increased (Figure 1D,E). Overall, USP7 knockout enhances the survival rate of LPS‐treated mice.

### 3.2 USP7 Deficiency Inhibits the Release of NETs in LPS‐Treated Mice

**FIGURE 2:**
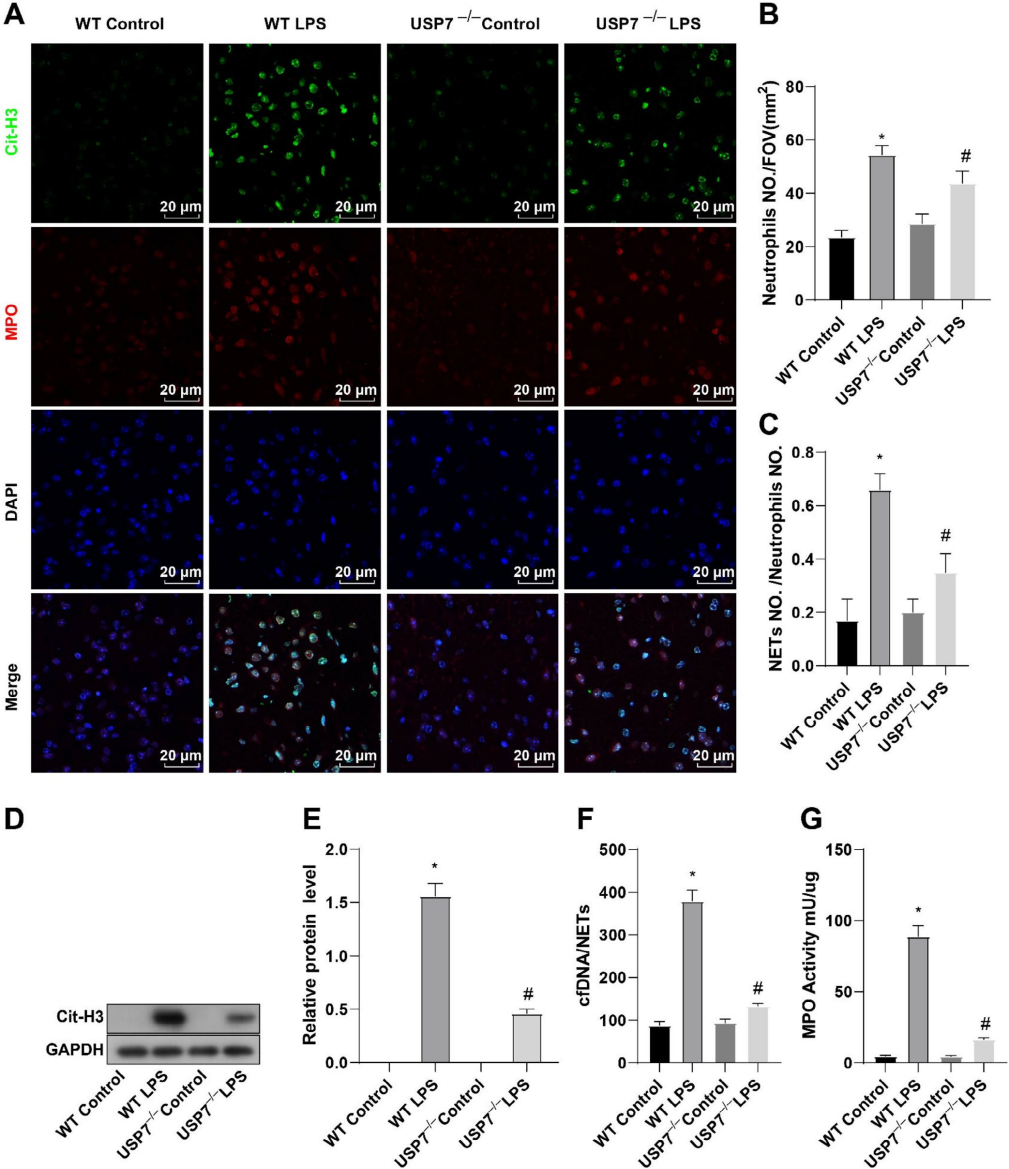
USP7 deficiency affects NET release in septic mice. (A–C) Immunofluorescence detection of NET expression in peripheral blood and corresponding bar graph. (C, D) Western blot analysis of Cit‐H3 expression levels in peripheral blood serum and bar graph. (E) Quantification of cfDNA/NETs in peripheral blood of mice. (G) Detection of MPO levels; *p < 0.05 compared to the WT Control group, ^#^ *p* < 0.05 compared to the WT LPS group; data were presented as mean ± SD; comparisons between groups were performed using one‐way ANOVA, *n* = 6.

The expression of NETs in peripheral blood was evaluated using immunofluorescence to ascertain the effect of USP7 on NET release in septic mice. The findings demonstrated an increase in NET fluorescence intensity in the WT LPS group compared to the WT Control group. In contrast, the USP7^−/−^ LPS group showed a decrease in NET fluorescence intensity compared to the WT LPS group (Figure 2A–C). Western blot analysis revealed that the WT LPS group showed higher Cit‐H3 expression in peripheral blood serum than the WT Control group, while the USP7^−/−^ LPS group exhibited lower Cit‐H3 expression than the WT LPS group (Figure 2D,E). Quantification of cfDNA/NET levels in the peripheral blood of mice showed that compared to the WT Control group, the WT LPS group had a significant increase in cfDNA/NET levels, while the USP7^−/−^ LPS group had lowered cfDNA/NET levels in contrast to the WT LPS group (Figure 2F). Additionally, MPO levels were higher in the WT LPS group than in the WT Control group but were lower in the USP7^−/−^ LPS group than in the WT LPS group (Figure 2G). Altogether, USP7 knockout depresses NET release in LPS‐treated mice.

### 3.3 Deficiency of USP7 Suppresses Multiorgan Dysfunction in LPS‐Treated Mice

**FIGURE 3:**
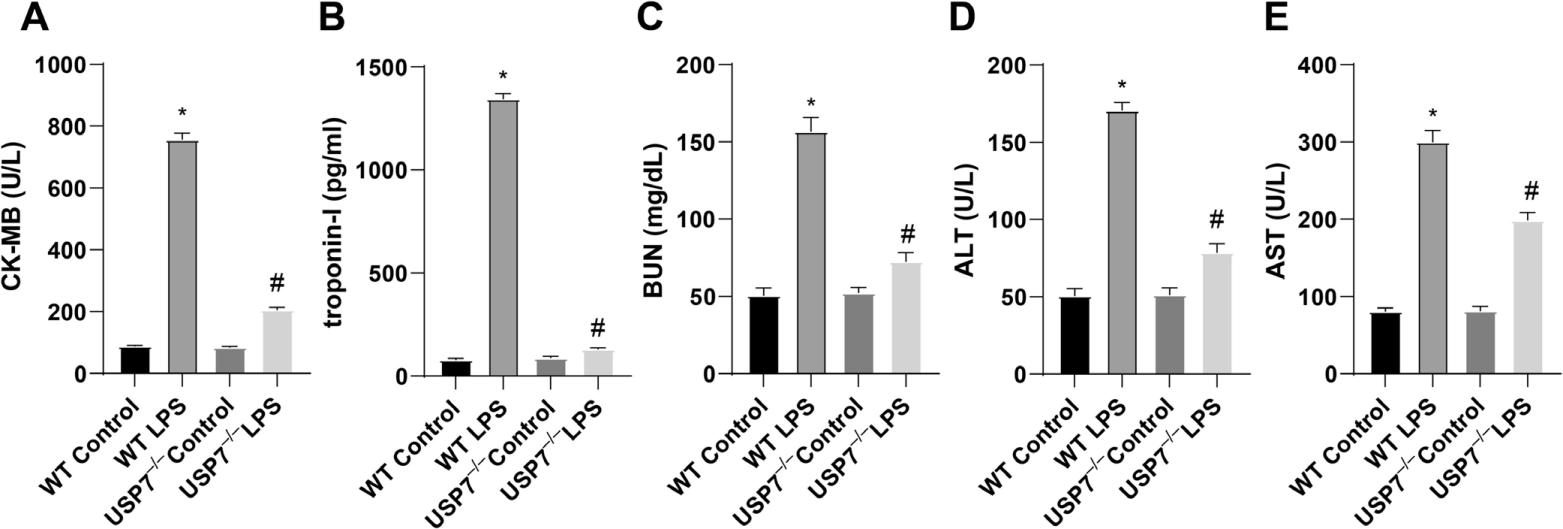
USP7 deficiency affects multiorgan dysfunction in septic mice. (A, B) Detection of cardiac injury biomarkers CK‐MB and troponin‐I using kits. (C) Detection of kidney injury biomarker BUN using kits. (D, E) Detection of liver injury biomarkers ALT and AST using kits; *p < 0.05 compared to the WT Control group, ^#^ *p* < 0.05 compared to the WT LPS group; data were presented as mean ± SD; comparisons between groups were performed using one‐way ANOVA; *n* = 6.

To clarify the role of USP7 in organ dysfunction during sepsis, we measured the expression of cardiac CK‐MB and troponin‐I. The results displayed that the WT LPS group exhibited higher expression of CK‐MB and troponin‐I than the WT Control group, whereas the USP7^−/−^ LPS group showed lower expression of CK‐MB and troponin‐I than the WT LPS group (Figure 3A,B). Next, we assessed kidney function by measuring BUN levels and analyzed liver function by detecting ALT and AST levels. The results revealed that BUN, ALT, and AST levels were enhanced in the WT LPS group compared to the WT Control group but were lowered in the USP7^−/−^ LPS group compared with the WT LPS group (Figure 3C–E).

**FIGURE 4:**
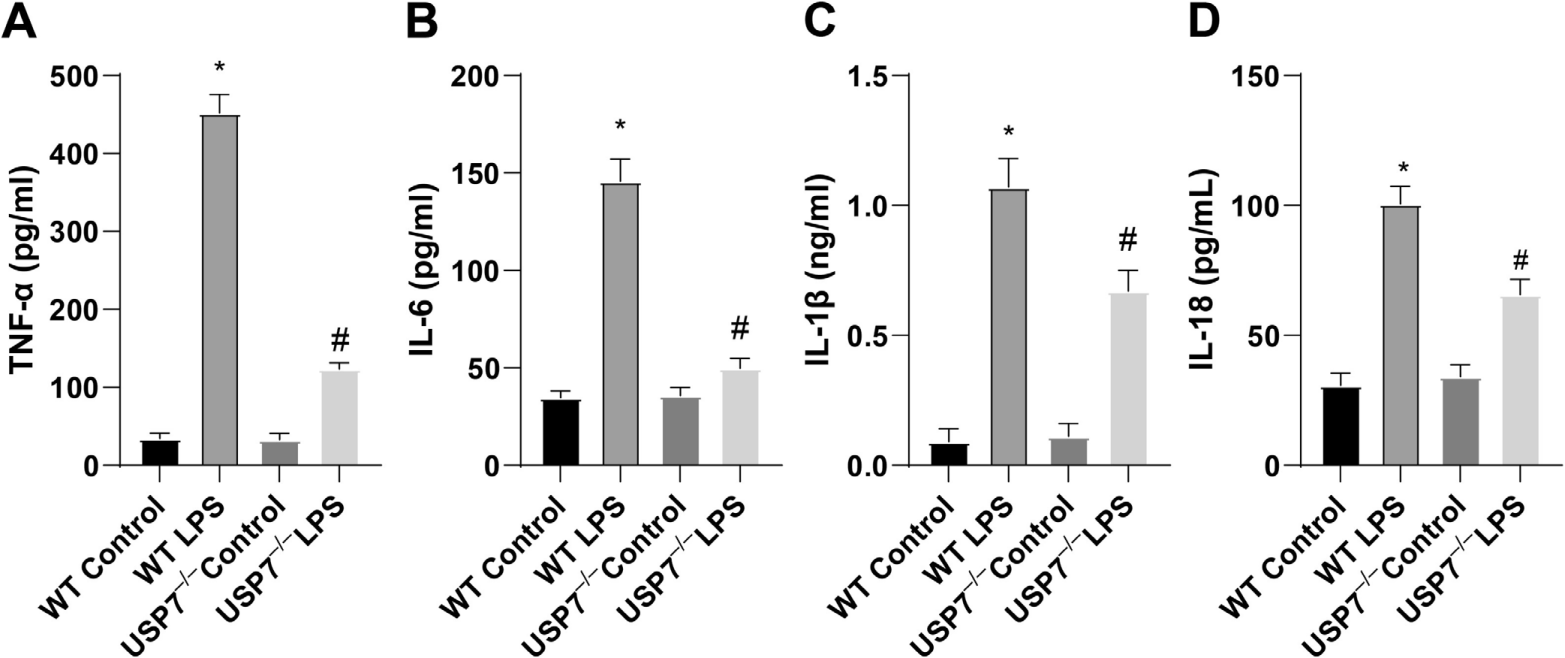
USP7 deficiency affects inflammation in septic mice. (A–D) ELISA measurement of inflammatory factors TNF‐α, IL‐1β, IL‐6, and IL‐8; *p < 0.05 compared to the WT Control group, ^#^ *p* < 0.05 compared to the WT LPS group; data were presented as mean ± SD; comparisons between groups were performed using one‐way ANOVA; *n* = 6.

According to ELISA data, the levels of inflammatory cytokines TNF‐α, IL‐1β, IL‐6, and IL‐8 were substantially higher in the WT LPS group than in the WT Control group but lower in the USP7^−/−^ LPS group than in the WT LPS group (Figure 4A–D). Collectively, USP7 knockout represses multiorgan dysfunction and inflammation in LPS‐treated mice.

### 3.4 Deficiency of USP7 Hinders Cellular Inflammation by Regulating ICAM‐1+ Neutrophils

**FIGURE 5:**
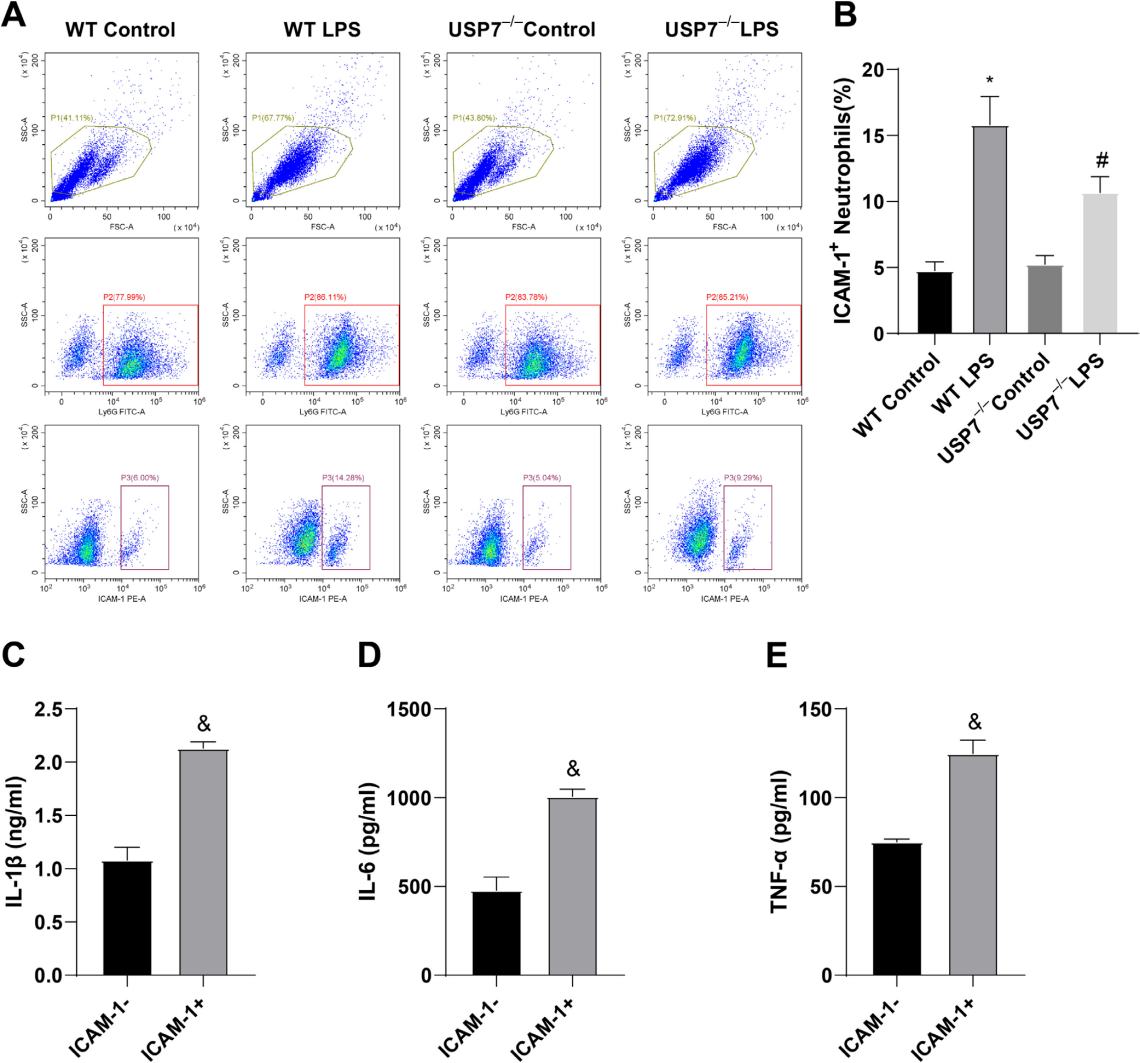
USP7 knockout affects cell inflammation by regulating ICAM‐1+ neutrophils. (A, B) Flow cytometry analysis of ICAM‐1+ neutrophil counts in each group and corresponding bar graph (P1: cells were gated based on size, intracellular granularity, and side scatter; P2: neutrophils were gated based on Ly6G‐positive staining to select the Ly6G‐positive population; P3: Ly6G‐positive cells were gated based on ICAM‐1 antibody staining to select the ICAM‐1‐positive population.). (C–E) ELISA measurement of TNF‐α, IL‐1β, and IL‐6 levels; *p < 0.05 compared to the WT control, ^#^ *p* < 0.05 compared to the WT LPS group, ^&^ *p* < 0.05 compared to the ICAM‐1‐ group; data were presented as mean ± SD; comparisons between groups were performed using one‐way ANOVA; *n* = 6; experiments were repeated three times.

NETs are primarily produced by ICAM‐1+ neutrophils. To investigate whether USP7 affects NETs and the inflammatory function of macrophages via ICAM‐1+ neutrophils, we used flow cytometry to assess the number of ICAM‐1+ neutrophils. The results showed that the WT LPS group had a higher number of ICAM‐1+ neutrophils than the WT Control group. The USP7^−/−^ LPS group exhibited a decrease in ICAM‐1+ neutrophils in contrast to the WT LPS group (Figure 5A,B). BMDMs from WT mice were then co‐cultured with FACS‐sorted ICAM‐1+ or ICAM‐1− neutrophils. ELISA data indicated higher levels of TNF‐α, IL‐1β, and IL‐6 in the ICAM‐1+ group than in the ICAM‐1− group (Figure 5C–E). In summary, USP7 knockout suppresses inflammation by regulating ICAM‐1+ neutrophils.

### 3.5 USP7 Stabilizes ICAM‐1 Expression Through Deubiquitination in Sepsis

**FIGURE 6:**
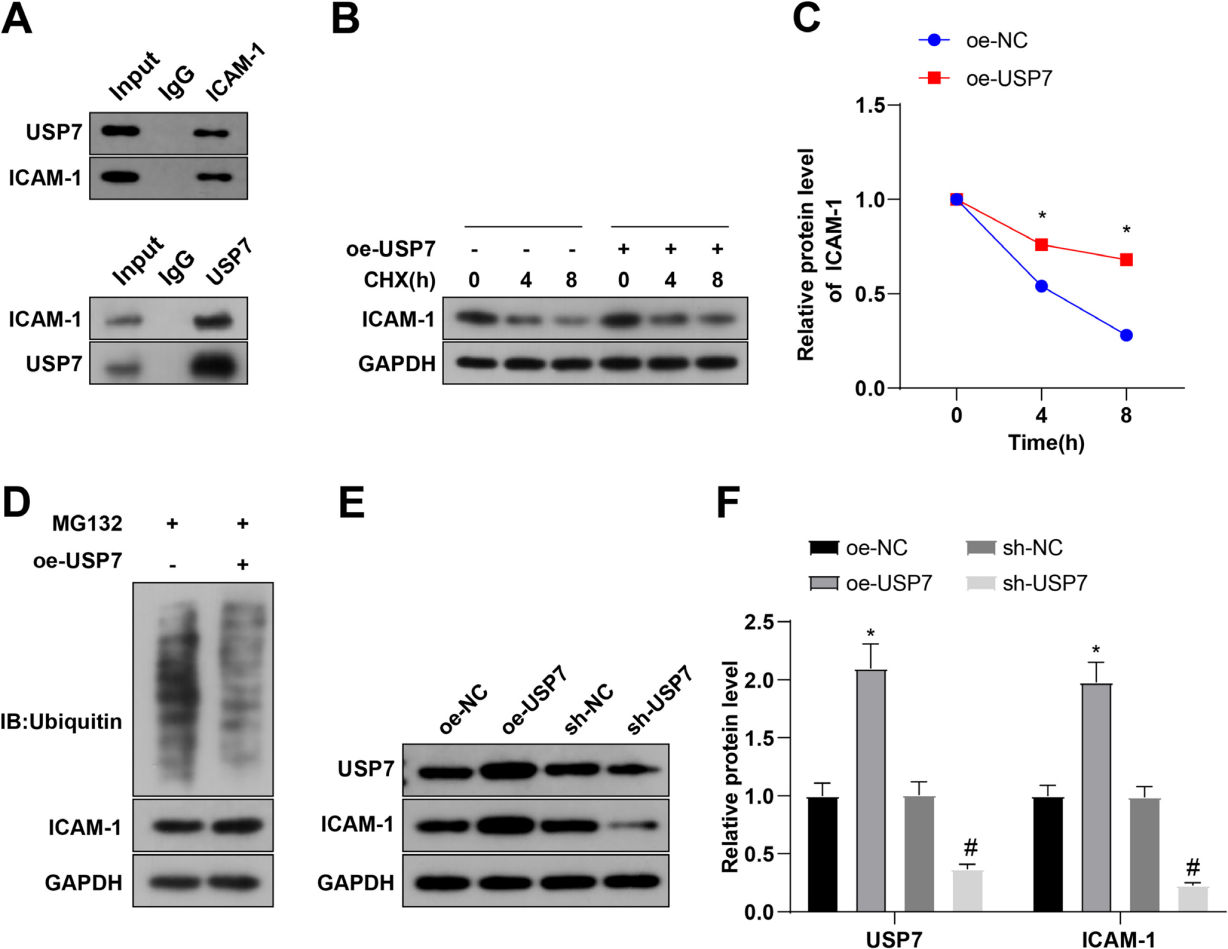
USP7 regulates ICAM‐1 expression in sepsis through deubiquitination. (A) Co‐IP detection of the interaction between ICAM‐1 and USP7 in neutrophils. (B, C) Western blot analysis of ICAM‐1 expression in neutrophils and corresponding bar graph (cycloheximide concentration: 100 μg/mL). (D) Western blot analysis of ICAM‐1 ubiquitination and expression in neutrophils (proteasome inhibitor MG132 concentration: 50 μg/mL). (E, F) Western blot analysis of USP7 and ICAM‐1 expression in neutrophils and corresponding bar graph; *p < 0.05 compared to the oe‐NC group, ^#^ *p* < 0.05 compared to the sh‐NC group; data were presented as mean ± SD; comparisons between groups were performed using the *t* test; experiments were repeated three times.

This study further clarified the regulatory mechanism between USP7 and ICAM‐1. Prediction with the UbiBrowser public database (http://ubibrowser.bio‐it.cn/ubibrowser_v3/) exhibited that ICAM‐1 might be stabilized by the deubiquitinase USP7. To confirm whether USP7 affects sepsis progression by interacting with ICAM‐1, Co‐IP assays were conducted. The data displayed that ICAM‐1 interacted with USP7 and mutually pulled down related proteins (Figure 6A). Protein stability tests revealed that overexpression of USP7 diminished ICAM‐1 protein expression in the presence of cycloheximide treatment, stabilizing ICAM‐1 expression (Figure 6B,C). Additionally, USP7 overexpression decreased ICAM‐1 ubiquitination levels (Figure 6D). Western blot demonstrated that oe‐USP7 markedly augmented USP7 and ICAM‐1 expression; compared with the sh‐NC group, USP7 and ICAM‐1 levels in the sh‐USP7 group were significantly decreased (Figure 6E,F). In conclusion, USP7 stabilizes ICAM‐1 expression through deubiquitination.

## 4 Discussion

Excessive NET formation has been implicated in the exacerbation of inflammatory injury and organ damage in septic patients, and prolonged and excessive inflammatory responses strongly correlate to the severity of the disease [19]. Consequently, understanding the mechanisms behind the pathophysiology of sepsis is essential for identifying potential therapeutic targets and improving patient outcomes. Although earlier research has reported that USP7 contributes to sepsis‐induced myocardial injury and cardiomyocyte pyroptosis through deubiquitination [9], its involvement in NET formation and broader inflammatory responses in sepsis remains poorly understood. Therefore, this study analyzed the mechanisms by which USP7 modulates NET formation and inflammation in sepsis and elaborated that USP7 knockout depressed NET formation and inflammation in sepsis by down‐regulating ICAM‐1.

USP7 has been revealed to participate in inflammation in diverse LPS‐induced models. For instance, USP7 positively modulates the expression of proinflammatory mediators in response to LPS exposure, and USP7 deficiency is linked to heightened susceptibility to infection [20]. Moreover, silencing USP7 mitigates inflammation and reduces cellular apoptosis in an acute lung injury model induced by intratracheal LPS administration [21]. More importantly, USP7 is upregulated in septic mice, and USP7 overexpression facilitates cardiac dysfunction and myocardial injury and augments IL‐1β, IL‐18, TNF‐α, and IL‐6 levels in myocardial tissues of septic mice [9]. In the present study, USP7 was knocked out in mice before the establishment of a mouse model of LPS‐induced sepsis to explore the function of USP7 in sepsis. The results revealed that USP7 knockout increased the survival rate of LPS‐treated mice. In addition, this study also demonstrated that USP7 knockout suppressed NET release in LPS‐treated mice. NETosis, a specialized form of cell death in activated neutrophils, results in the release of NETs, which are web‐like structures composed of DNA and antimicrobial proteins, such as MPO, neutrophil elastase, and histone [22]. Proinflammatory cytokines, including TNF‐α, IL‐1β, IL‐6, and IL‐8, are key mediators of neutrophil activation and NET formation [23]. In the present study, a significant reduction in MPO expression and TNF‐α, IL‐1β, IL‐6, and IL‐8 levels was noted in USP7^−/−^ mice treated with LPS, suggesting diminished NET release. Similarly, USP47 inhibition was shown to reduce both NET formation and the levels of proinflammatory cytokines in myocardial infarction [24]. Furthermore, the pathological injury observed in the myocardium and infarcted areas is correlated with elevated levels of cardiac biomarkers, such as CK‐MB and troponin‐I [25]. A prior study displayed that USP7 upregulation augmented troponin‐I and CK‐MB expression in hypoxia‐induced cardiomyocyte injury [26]. Consistently, our results elucidated that USP7 knockout markedly reduced CK‐MB and troponin‐I expression in LPS‐treated mice, underlining the protective effect of USP7 inhibition on cardiac function. Additionally, our data exhibited that USP7 knockout led to a decrease in BUN, ALT, and AST levels, which are commonly used markers for liver and kidney function [27].

As a deubiquitinating enzyme, USP7 mediates the stability of multiple proteins involved in numerous cell processes, including DNA damage response, epigenetic control of gene expression, and transcription [28]. For instance, USP7 deubiquitinates and stabilizes LATS1/2 in sepsis‐induced acute lung injury [29]. Innovatively, our mechanistic analysis revealed that USP7 stabilized ICAM‐1 through deubiquitination and that USP7 knockout depressed NET formation and inflammation in LPS‐treated mice by downregulating ICAM‐1. Activation of proinflammatory responses was evident not only through elevated levels of TNF‐α and IL‐1β but also by a significant upregulation of ICAM‐1 [30]. A former study documented that ICAM‐1‐expressing neutrophils produced excessive amounts of NETs, further amplifying the inflammatory cascade in sepsis [12]. Moreover, another study exhibited that ICAM‐1 knockout in neutrophils mitigated inflammation in sepsis by lowering TNF‐α and IL‐6 levels [12]. Additionally, Zhu et al. observed a marked increase in the concentration of ICAM‐1 in both the liver and lungs of septic mice, as well as its expression on vascular endothelial cells in these tissues [31]. Notably, USP7 knockout reduced ICAM‐1+ neutrophil expression. WT mouse BMDMs were co‐cultured with FACS‐sorted ICAM‐1+ or ICAM‐1− neutrophils. Compared with the ICAM‐1− group, TNF‐α, IL‐1β, and IL‐6 expression were significantly increased in the ICAM‐1+ group.

In conclusion, our findings provide novel insights into the specific role of USP7 in sepsis from the perspective of its regulation of ICAM‐1 expression and its subsequent effects on NET formation and inflammation. Our data unveiled that USP7 knockout declined ICAM‐1 expression, exerting suppressive effects on NET formation and inflammation in LPS‐treated mice. However, although we observed the significant effects of USP7 in the mouse model, these findings have not been validated in clinical settings. Therefore, the generalizability of these findings to human sepsis requires careful validation in clinical settings.

## Ethics Statement

All animal experiments rigorously followed “Guidelines on Humane Treatment of Laboratory Animals” issued by the Ministry of Science and Technology of China and the Guide for the Care and Use of Laboratory Animals issued by NIH. Additionally, the study strictly adhered to the principle of completing the experiment with the minimum number of animals and minimizing the suffering of the experimental animals. The animal experiments have been carried out in accordance with the ARRIVE guidelines (ethical approval number: SH9H‐2025‐A1212‐SB).

## Conflicts of Interest

The authors declare no conflicts of interest.

## Data Availability

The data that support the findings of this study are available from the corresponding author upon reasonable request.
